# The neurological pathology of peroxisomal ACBD5 deficiency – lessons from patients and mouse models

**DOI:** 10.3389/fnmol.2025.1602343

**Published:** 2025-07-02

**Authors:** Michael L. Dawes, Jim P. Haberlander, Markus Islinger, Michael Schrader

**Affiliations:** ^1^Department of Biosciences, Faculty of Health and Life Sciences, University of Exeter, Exeter, United Kingdom; ^2^Institute of Neuroanatomy, Medical Faculty Mannheim, Heidelberg University, Mannheim, Germany

**Keywords:** peroxisomes, ACBD5, VAP, membrane contact sites, RDLKD, fatty acid metabolism

## Abstract

The absence or dysfunction of the peroxisomal membrane protein Acyl-CoA Binding Domain-Containing Protein 5 (ACBD5) is the cause of the most recently discovered peroxisomal disorder “Retinal Dystrophy with Leukodystrophy” (RDLKD). ACBD5 is a tail-anchored protein, anchored by its C-terminus into the peroxisomal membrane; hence, the bulk of its amino acid sequence faces the cytosol. With respect to ACBD5’s molecular functions, RDLKD is unique since it is not only an accessory protein for the import of very-long-chain fatty acids (VLCFAs) into peroxisomes but also the first identified peroxisomal tethering protein facilitating membrane contacts with the endoplasmic reticulum (ER). Consequently, RDLKD is neither a peroxisomal biogenesis disorder nor single enzyme deficiency, since a deficiency in ACBD5 likely affects several aspects of peroxisomal function including VLCFA degradation, ether lipid synthesis, docosahexaenoic acid synthesis but also the transfer of membrane lipids from the ER to peroxisomes. Hence, RDLKD appears to be a multifactorial disorder leading to a mosaic pathology, combining symptoms caused by the disruption of several pathways. In this review, we will highlight recent findings obtained from case reports of RDLKD patients as well as insights from ACBD5-deficient mouse models to better understand its complex retinal and brain pathology. Moreover, we will discuss the possible contribution of the different dysregulated metabolites in the neurological pathogenesis of this latest peroxisomal disorder.

## 1 Introduction

### 1.1 The role of peroxisomes in lipid metabolism and neuropathology

**FIGURE 1:**
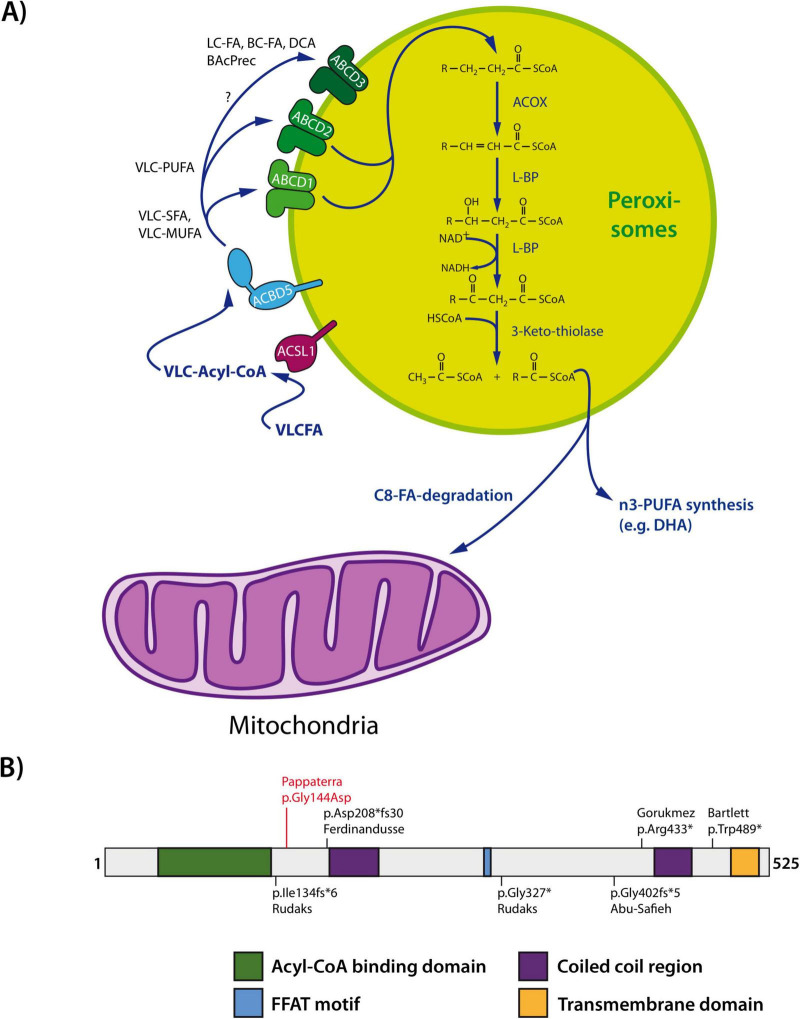
**(A)** Overview of peroxisomal very-long-chain fatty acid (VLCFA) β-oxidation. Long chain fatty acid- CoA ligase (ACSL1) activates VLCFAs generating VLC-acyl-CoA. ACBD5 can capture VLC-acyl-CoA via its acyl-CoA binding domain and hands it over to the peroxisomal ABC transporters for VLCFAs (e.g., ABCD1 and ABCD2). VLCFAs are then imported into the peroxisomal matrix and degraded by the peroxisomal β- oxidation pathway in four consecutive steps: (i) dehydrogenation catalyzed by acyl-CoA oxidase (ACOX), (ii) hydration and (iii) second dehydrogenation mediated by L-bifunctional protein (L-BP) with enoyl-CoA hydratase and 3-hydroxyacyl-CoA dehydrogenase activity, and (iv) thiolytic cleavage by 3-ketothiolase. The chain-shortened acyl-CoAs can be routed to mitochondria for complete degradation via the mitochondrial β-oxidation pathway or used for the synthesis of omega-3 polyunsaturated fatty acids (PUFAs) such as docosahexaenoic acid (DHA). **(B)** ACBD5 domain structure. Known mutations are indicated. FFAT, two phenylalanines (FF) in an acidic tract (adapted from Schrader et al., 2020). BAcPrec, bile acid precursors; BC, branched-chain; DCA, dicarboxylic acid; FA, fatty acid; LC, long-chain, MUFA, monounsaturated fatty acid; SFA, saturated fatty acid; VLC, very-long-chain.

Peroxisomes are oxidative organelles with key functions in cellular redox homeostasis and lipid metabolism. Mammalian peroxisomes harbor a fatty acid β-oxidation pathway, which is essential for the degradation of a variety of fatty acid substrates including very-long-chain fatty acids (VLCFAs), branched-chain fatty acids such as pristanic acid, bile acid intermediates, long-chain dicarboxylic acids (DCAs), eicosanoids, and the side chains of certain xenobiotics, which can solely be degraded in peroxisomes (Wanders et al., 2023; Vaz et al., 2025; Figure 1 for overview). However, mammalian peroxisomes only chain-shorten fatty acids and need to route them to mitochondria for full oxidation via the mitochondrial β-oxidation pathway (Schrader et al., 2015). Notably, the key enzyme in peroxisomal fatty acid β-oxidation is an acyl-CoA oxidase, which generates hydrogen peroxide. The latter is decomposed by peroxisomal catalase but can also act as an important signaling molecule (Fransen and Lismont, 2024). Mammalian peroxisomes are also involved in the synthesis of lipids, in particular ether phospholipids and plasmalogens (Wanders et al., 2023). Peroxisomes perform the first steps in ether lipid synthesis, as they contain the enzyme for ether bond formation, but cooperate metabolically with the endoplasmic reticulum (ER), where ether lipid biosynthesis is completed. Furthermore, they are involved in the synthesis of polyunsaturated fatty acids such as docosahexaenoic acid [DHA, C22:6(n-3)], which is generated from a C24-precursor by one cycle of peroxisomal β-oxidation (Figure 1).

Defects in single enzymes of these metabolic pathways or in the biogenesis of peroxisomes, which usually result in a loss of all metabolic functions, can lead to severe disorders with developmental and neurological abnormalities (Steinberg et al., 2020; Wanders et al., 2023). The latter include motor and sensory functions such as hearing loss, and are characterized by demyelination, inflammatory processes, and neurodegeneration (Berger et al., 2016). Neurological abnormalities are caused by the accumulation of peroxisomal substrates, particularly VLCFAs, which have been shown to disturb the membrane organization of axons (Kleinecke et al., 2017; Szrok-Jurga et al., 2023). Furthermore, there is a shortage of peroxisomal lipid products such as ether phospholipids, which are important components of myelin sheaths and other cellular membranes. DHA is a crucial constituent in the brain and retina (Wanders et al., 2023). Inflammatory processes can be caused by altered signaling of macrophages and infiltration of immune cells in the brain (Berger et al., 2016; Fransen et al., 2020; Zierfuss et al., 2022). Furthermore, a role of peroxisomes in oxidative stress and redox imbalance in neurodegenerative diseases has been reported (Fransen et al., 2020).

Fatty acid substrates are imported into mammalian peroxisomes by three ABC transporters of the superfamily D (ABCD1–3) (Figure 1). ABCD1 and ABCD2, which encode the adrenoleukodystrophy protein ALDP and ALDP-like protein, respectively, show distinct substrate specificities for VLCFAs. Defects in ABCD1 cause adrenoleukodystrophy, one of the most prominent peroxisomal single enzyme deficiencies (PEDs), which is caused by an accumulation of VLCFAs resulting in leukodystrophy and adrenal insufficiency (Engelen, 2024; Bougnères and Le Stunff, 2025; Vaz et al., 2025), whereas patients with a defective ABCD2 have not yet been identified.

### 1.2 Properties and molecular functions of ACBD5

Recently, a role for the tail-anchored peroxisomal membrane protein ACBD5 as an accessory protein in VLCFA β-oxidation has been revealed (Ferdinandusse et al., 2017; Yagita et al., 2017; Schrader et al., 2020; Figure 1). ACBD5 belongs to a large multigene family of acyl-CoA binding domain containing proteins, comprised of soluble proteins, multifunctional enzymes, and membrane proteins (Islinger et al., 2020). ACBD5 has been identified as a peroxisomal protein in several proteomics studies (Kikuchi et al., 2004; Islinger et al., 2007; Wiese et al., 2007). Its peroxisomal targeting is mediated by physicochemical parameters of the transmembrane domain and tail sequence, and involves PEX19, the peroxisomal import receptor/chaperone for peroxisomal membrane proteins (Costello et al., 2017a).

#### 1.2.1 The peroxisome-ER tethering function of ACBD5

**FIGURE 2:**
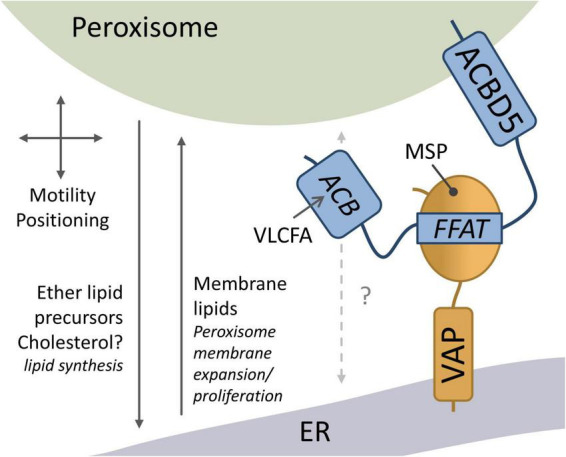
Overview of ACBD5 cellular functions. The FFAT motif of peroxisomal ACBD5 interacts with the major sperm protein (MSP) domain of ER-resident VAP to mediate peroxisome (PO)-ER membrane contacts. The PO-ER membrane contacts have been implicated in peroxisome motility and positioning, the transfer of ether lipid/plasmalogen precursors for further synthesis in the ER, and the transfer of membrane lipids for peroxisome membrane expansion and proliferation. ACBD5 possesses an acyl-CoA binding (ACB) domain which can capture very-long-chain acyl CoA (VLCFA) for peroxisomal β-oxidation (adapted from Kors et al., 2022b).

Besides its acyl-CoA binding domain, ACBD5 contains a coiled-coil domain, which mediates homo-oligomerization, and a FFAT-like motif [two phenylalanines (FF) in an acidic tract] (Figure 1B). The latter has been shown to interact with the major sperm protein (MSP)-domain of ER-resident vesicle-associated membrane protein (VAMP)-associated protein (VAP). Both ACBD5 and VAP function as tether proteins linking the peroxisomes to the ER thus creating membrane contact sites (MCSs) (Costello et al., 2017b; Hua et al., 2017; Figures 1, 2). Co-expression of ACBD5 and VAPB results in an increase of peroxisome (PO)-ER contacts, whereas loss of ACBD5 or VAP decreases PO-ER contacts as revealed by quantitative electron microscopy. The formation of ACBD5-VAP mediated PO-ER contacts can be regulated by phosphorylation of the ACBD5 FFAT-like motif; phosphorylation of serine 269 in the core region of the FFAT-motif of human ACBD5 results in a loss of VAPB binding and reduction in PO-ER contacts. Phosphorylation of Ser269 can be mediated by GSK3β (Kors et al., 2022a).

Besides ACBD5, another tail-anchored peroxisomal protein, ACBD4, has been identified (Costello et al., 2017a; Costello et al., 2017c). ACBD4 also contains a FFAT-like motif and can interact with ER-resident VAP to mediate PO-ER contacts. When overexpressed in ACBD5 knock out cells, ACBD4 can compensate for the loss of ACBD5 and restore PO-ER MCSs (Costello et al., 2023). However, ACBD5 appears to be the major PO-ER tether, and ACBD4 may fulfill other, potentially regulatory functions (Costello et al., 2023). All vertebrates possess an ACBD5 and ACBD4 gene; the latter has likely been generated by gene duplication of ACBD5. In contrast, invertebrates and fungi only encode a single ACBD4/5-like protein (Kors et al., 2024).

With respect to the functions of PO-ER MCSs, it has been revealed that they determine the positioning of peroxisomes and restrict peroxisomal mobility (Figure 2). Silencing of ACBD5 in human fibroblasts caused increased displacement of peroxisomes (Costello et al., 2017b). Overexpression of ACBD5 in hippocampal neuron cultures changed motility and positioning (Wang et al., 2018). The PO-ER MCSs likely contribute to the uniform distribution of peroxisomes observed in many cell types. It has recently been shown that loss of the ACBD4/5-like protein in *Drosophila melanogaster* (*Dm*) results in a displacement of peroxisomes in axons in the fly wing, with an increase in numbers compared to control flies (Kors et al., 2024). *Dm*_ACBD4/5 targets peroxisomes and contains a FFAT motif, which is required for the interaction with Dm_Vap33, the VAP ortholog of *Drosophila*.

Furthermore, ACBD5-VAP bring both the ER and peroxisomes in close proximity to enable phospholipid transfer from the ER to peroxisomes for membrane expansion, a pre-requisite for division and multiplication of peroxisomes (Figure 2; Costello et al., 2017b; Darwisch et al., 2020; Carmichael and Schrader, 2022).

#### 1.2.2 ACBD5 function as an accessory protein in VLCFA β-oxidation

**FIGURE 3:**
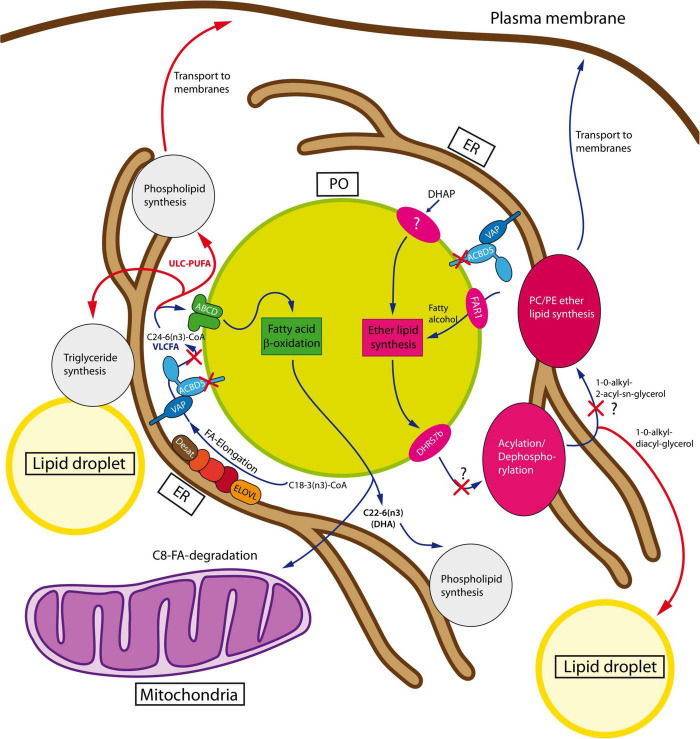
Peroxisomal metabolic interaction with the ER. The ether lipid synthesis pathway (shown in magenta) is initiated in peroxisomes by acylation of dihydroxyacetone phosphate (DHAP). In/at peroxisomes, 1-acyl-DHAP is converted stepwise to 1-alkyl-DHAP and 1-alkyl-2-hydroxy-glycerophosphate (GPA), which must be transferred to the ER for completion of the pathway. At the ER, GPA is supplied with an acyl-chain at the *sn2*-position and a phosphatidylethanolamine (PE) or phosphatidylcholine (PC) head group to yield ether phospholipids. Alternatively, the ether lipids can receive a second acyl group at *sn3* to be stored in lipid droplets. Consequently, a disruption of the pathway might lead to GPA accumulation, which has not yet been experimentally analyzed. Peroxisomal β-oxidation is required for the synthesis of *n3*-polyunsaturated fatty acids like DHA (pathway in green). At the ER, α-linolenic acid is elongated and desaturated to tetracosahexaenoic acid (C24:6n-3), which must be delivered to peroxisomes to be shortened to DHA (C22:6n3) in one single round of β-oxidation. Loss of the ACBD5-mediated tethering complex could presumably lead to local enrichment of C24:6n3 at the ER to be further elongated and desaturated by the ER fatty acid elongation system. This could lead to the accumulation of VLC-PUFA to be finally incorporated into phospholipids or triglycerides (adapted from Darwisch et al., 2020). For details see text. FA, fatty acid; PUFA, polyunsaturated fatty acid; UCL, ultra-long-chain.

Tethering and MCS formation have been suggested to create a lipid hub at the PO-ER interface, which allows regulation of the metabolism of VLCFA (Schrader et al., 2020; Figure 3). In line with the observed reduction of peroxisomal β-oxidation in RDLKD patient fibroblasts (see section “2.4 Biochemical and cellular alterations in ACBD5-deficient patients”), the acyl-CoA binding domain of ACBD5 has been shown to bind very long-chain acyl-CoA (VLC-CoA) with high affinity (Yagita et al., 2017; Costello et al., 2023). This suggests that ACBD5 may sequester the low-abundance VLC-CoA from the cytosol, thereby increasing their local concentration near peroxisomes and enhancing their import efficiency by routing them to the ABCD1 or ABCD2 transporters for uptake into peroxisomes. Supporting this idea, ACBD5 has been reported to interact with the acyl-CoA synthetase ACSL1, implying that VLCFAs may be directly transferred to ACBD5 immediately after activation, further streamlining their import into peroxisomes (Young et al., 2018).

VLCFAs can be synthesized from long-chain fatty acids by elongases localized at the ER membrane; however, inefficient β-oxidation can lead to their accumulation at the ER membrane, which results in further elongation and lipid toxicity (Kemp et al., 2005). To prevent this, ACBD5 may also regulate VLCFA uptake into peroxisomes at the PO-ER interface, promoting uptake and subsequent β-oxidation when there is a surplus, or reduce it, when more elongated fatty acids are required, e.g., for phospholipid synthesis at the ER (Figure 3). This likely explains why a tethering function has been combined with the ability to bind VLC-CoAs via the acyl-CoA binding domain.

In recent years, increasing numbers of patients with mutations in *ACBD5* have been identified, while no patients with mutations in *ACBD4* have been reported to date. In the following sections, we will focus on the pathophysiology of ACBD5 deficiency.

## 2 Symptomatology of patients with ACBD5 deficiency (RDLKD)

ACBD5 deficiency, referred to in the OMIM database as “retinal dystrophy with Leukodystrophy” (RDLKD), is a rare congenital disorder caused by the absence of the peroxisomal membrane protein ACBD5. As its OMIM designation suggests, patients typically present with neurological symptoms, aligning with the broader clinical phenotype observed in many peroxisomal disorders. However, according to its dual role in VLCFA import and membrane tethering, RDLKD may present with a pathology deviating from typical single enzyme deficiencies of peroxisomal β-oxidation (see section “1.2 Properties and molecular functions of ACBD5”; Figures 1–3). Thus, a detailed examination of the clinical features of reported cases is essential to appreciate the unique nature of this recently characterized peroxisomal disorder. To date, eight publications have reported on 16 patients with ACBD5 deficiency (Abu-Safieh et al., 2013; Ferdinandusse et al., 2017; Bartlett et al., 2021; Gorukmez et al., 2022; Pappaterra-Rodriguez et al., 2022; Hasturk et al., 2024; Rudaks et al., 2024; Al Shamsi et al., 2025; Supplementary Table 1). Among these, 14 patients carry homozygous variants in the ACBD5 gene, comprising 10 distinct mutations—all resulting in frameshifts and, consequently, a complete loss of ACBD5 protein expression (Figure 1B). Two patients harbor a heterozygous c.431G>A (p.Gly144Asp) point mutation in *ACBD5* (Pappaterra-Rodriguez et al., 2022) and therefore do not formally meet the diagnostic criteria for RDLKD (Figure 1B). Nonetheless, as the only individuals reported to express a mutant form of ACBD5, their phenotypes merit inclusion in this review. As anticipated, these two heterozygous patients exhibited a significantly milder clinical course, presenting solely with ophthalmological symptoms. In contrast, all patients with homozygous mutations experienced a more severe disease trajectory, characterized by both neurological impairment and progressive vision loss.

Symptomatic manifestations of the c.431G>A (p.Gly144Asp) mutation in the older of the two reported patients, a 48-year-old male, included night blindness, dyschromatopsia, and progressive vision loss. Based on these clinical features, he was initially diagnosed with retinitis pigmentosa, a condition also associated with the peroxisomal Refsum disease spectrum (Wanders et al., 2001). His 18-year-old daughter, who carries the same *ACBD5* mutation, did not report any visual impairment; however, ophthalmological examination revealed incipient atrophy of the central fovea. Notably, neither patient exhibited neurological symptoms (Pappaterra-Rodriguez et al., 2022; Supplementary Table 1). At the molecular level, glycine 144, the residue affected by this mutation, is highly conserved across animal species and is located within a conserved α-helix adjacent to the acyl-CoA binding domain (Kors et al., 2024). Future studies are needed to determine whether the substitution to asparagine at this position leads to a gain of function—potentially by altering interactions with wild-type ACBD5—or whether a reduction in functional ACBD5 due to heterozygosity is sufficient to cause a pathological phenotype. Regardless, the presence of clinical symptoms in heterozygous carriers suggests that specific ACBD5 point mutations may be more prevalent and clinically relevant than currently recognized but remain underdiagnosed due to their mild or isolated symptomatology.

**FIGURE 4:**
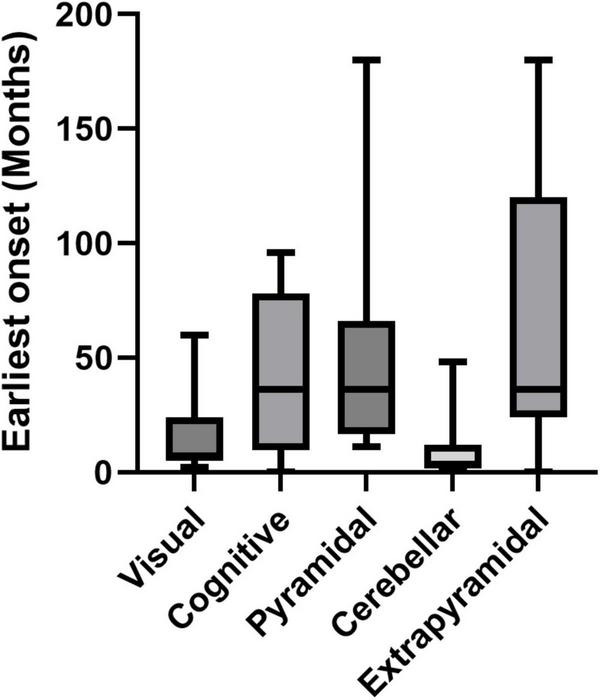
Graph depicting variability of the earliest onset of symptoms in patients. Note that cerebellar and visual dysfunction commonly occur as first signs for an ACBD5-deficiency. “Extrapyramidal” excludes motor dysfunctions, which can be related to a cerebellar degeneration.

The remaining homozygous patients, all of whom lack functional ACBD5 protein expression, typically present with both ophthalmological and neurological symptoms shortly after birth. These symptoms progressively worsen over time, leading to a severe clinical picture that profoundly impacts daily life (Figure 4). In addition to this shared core pathology, affected individuals also exhibit a variety of other symptoms, with significant variability in their manifestation across patients (Supplementary Table 1). To better delineate the common clinical features of RDLKD and distinguish them from more infrequent or variable manifestations, a detailed analysis of the symptomatology observed in all reported cases will be provided in the following paragraphs.

### 2.1 Ophthalmological symptoms – a hallmark of ACBD5 deficiency?

Membrane lipids in the retina are particularly rich in DHA and VLCFA, both of which rely on peroxisomal β-oxidation for their synthesis and/or degradation (McMahon and Kedzierski, 2010; Swinkels and Baes, 2023). Hence, the retinal phenotype observed in ACBD5-deficient patients highlights the protein’s critical role in VLCFA metabolism (Yagita et al., 2017). Photosensitivity and photophobia are early symptoms of ACBD5 deficiency, reported in 8 out of 14 patients (Ferdinandusse et al., 2017; Bartlett et al., 2021; Gorukmez et al., 2022; Hasturk et al., 2024; Rudaks et al., 2024; Al Shamsi et al., 2025), with symptoms developing as early as 2 months (Rudaks et al., 2024) and as late as 2 years (Al Shamsi et al., 2025), reflecting the early onset of the retinal pathology (Figure 4 and Supplementary Table 1). Progressive vision loss, reported in 11 patients (Ferdinandusse et al., 2017; Bartlett et al., 2021; Gorukmez et al., 2022; Hasturk et al., 2024; Rudaks et al., 2024; Al Shamsi et al., 2025) appears to be one of the hallmarks of ACBD5 deficiency. Rod-cone or cone-rod dystrophies, affecting both photoreceptor types, were described in at least nine patients (Abu-Safieh et al., 2013; Ferdinandusse et al., 2017; Bartlett et al., 2021; Rudaks et al., 2024; Al Shamsi et al., 2025), distinguishing ACBD5 deficiency from pure macular dystrophies. However, signs of a conspicuous macular involvement—such as pale or atrophic maculae—were also seen in six cases (Hasturk et al., 2024; Al Shamsi et al., 2025), suggesting significant cone cell mortality. Indeed, optical coherence tomography images of the patient described by Hasturk et al. (2024) reveal pronounced degeneration of the outer layers of the central fovea, while the peripheral regions remain relatively preserved. This pattern suggests that cone photoreceptor cells are particularly vulnerable to the metabolic changes caused by ACBD5 deficiency. Nevertheless, monochromacy or compromised color vision were noted in only one case (Rudaks et al., 2024). Optic disc pallor, observed in six patients, additionally suggests ganglion cell degeneration, and retinal vasoconstriction, another feature shared with other retinodystrophies, was also reported in six patients (Bartlett et al., 2021; Al Shamsi et al., 2025). Pathological abnormalities of the retinal pigment epithelium (RPE)—which plays a critical role in degrading the distal ends of photoreceptor outer segments (POS) and selectively transporting metabolites from the underlying choroidea to photoreceptor cells—were observed in six patients (Bartlett et al., 2021; Gorukmez et al., 2022; Hasturk et al., 2024; Al Shamsi et al., 2025). It is possible that elevated VLCFA levels in POS membrane discs may lead to their accumulation within RPE cells, potentially disrupting RPE lipid metabolism and contributing to disease pathology.

In some cases, retinal degeneration was accompanied by myopia (three patients) (Al Shamsi et al., 2025), astigmatism (four patients) (Al Shamsi et al., 2025), or oculomotor dysfunction (three patients) (Ferdinandusse et al., 2017; Hasturk et al., 2024; Rudaks et al., 2024). However, given the limited number of reported cases, it remains unclear whether these features are directly related to ACBD5 deficiency or occur coincidentally in a subset of patients.

In four individuals, the ophthalmologic presentation was clinically diagnosed as retinitis pigmentosa (Bartlett et al., 2021; Gorukmez et al., 2022; Al Shamsi et al., 2025), a syndrome previously linked to other inherited peroxisomal disorders (Karuntu et al., 2024). In contrast, funduscopic visualizations presented by Hasturk et al. (2024) show vital papillae with regular vascularization and therefore provide no clear indications for a retinitis pigmentosa. Hence, further patient evaluations and future studies are needed to determine whether ACBD5-associated retinal dystrophy falls within the retinitis pigmentosa spectrum or represents a distinct pathological entity. In contrast to the retinal pathology observed in Zellweger spectrum patients—characterized by schisis-like, cystoid maculopathy with variable and severe central and peripheral degeneration—RDLKD presents a distinct pathology, marked by a conspicuous macular involvement. As the patient cohort is currently limited, it remains to be determined, if retinal alterations are indeed a hallmark of ACBD5 deficiency, and larger cohorts are required to define the phenotype variability of the RDLKD eye pathology.

### 2.2 Neurological symptoms of ACBD5-deficient patients

The complex neurological symptoms observed in ACBD5-deficient patients likely reflect dysfunction across multiple regions of the central nervous system (CNS). Among these, motor impairments are particularly prominent. All 14 reported patients exhibited reduced psychomotor abilities, which progressed to a complete inability to walk in the following years (Abu-Safieh et al., 2013; Ferdinandusse et al., 2017; Bartlett et al., 2021; Gorukmez et al., 2022; Hasturk et al., 2024; Rudaks et al., 2024; Al Shamsi et al., 2025; Supplementary Table 1).

#### 2.2.1 Cerebellar dysfunction

In parallel with the progressive decline in motor function, symptoms associated with damage to the cerebellum—such as nystagmus, ataxia, and intention tremor (Bodranghien et al., 2016)—were observed early in life (Figure 4). Nystagmus was diagnosed in 11 patients (Ferdinandusse et al., 2017; Bartlett et al., 2021; Gorukmez et al., 2022; Hasturk et al., 2024; Rudaks et al., 2024; Al Shamsi et al., 2025), with an early onset ranging from 1 month (Bartlett et al., 2021) to 4 years of age (Ferdinandusse et al., 2017; Supplementary Table 1). Intention tremor and ataxia were later identified in three and six patients, respectively (Ferdinandusse et al., 2017; Gorukmez et al., 2022; Rudaks et al., 2024; Al Shamsi et al., 2025).

Evidence of cerebellar dysfunction was also apparent in MRI scans, which revealed cerebellar atrophy in four patients (Bartlett et al., 2021; Hasturk et al., 2024; Rudaks et al., 2024; Al Shamsi et al., 2025). Additionally, demyelination of the cerebellar peduncles—including the lower (Ferdinandusse et al., 2017), middle (Gorukmez et al., 2022), and upper segments (Al Shamsi et al., 2025)—was reported. It remains unclear whether metabolic disruption of oligodendrocytes and subsequent axon demyelination precede cerebellar atrophy, or if primary degeneration of cerebellar neurons or glial cells initiates neuroinflammatory processes that ultimately lead to demyelination.

#### 2.2.2 Leukodystrophy and degeneration of CNS motor and sensory axon tracts

In addition to cerebellar peduncle demyelination, a widespread CNS leukodystrophy has been observed in all reported ACBD5-deficient patients (Supplementary Table 1). The leukodystrophy affected either the entire brain white matter (Rudaks et al., 2024), or only several distinct regions. Leukodystrophy of the deep white matter was identified in four patients (Ferdinandusse et al., 2017; Gorukmez et al., 2022; Al Shamsi et al., 2025), while periventricular white matter involvement was noted in five patients (Bartlett et al., 2021; Gorukmez et al., 2022; Al Shamsi et al., 2025). Additionally, posterior white matter changes were reported in three patients (Hasturk et al., 2024; Al Shamsi et al., 2025). As the brain’s largest commissural structure, the corpus callosum showed signs of demyelination on MRI in five cases (Gorukmez et al., 2022; Al Shamsi et al., 2025). Subcortical U-fiber demyelination was specifically reported in one case (Bartlett et al., 2021), whereas in four other patients, these regions appeared unaffected (Ferdinandusse et al., 2017; Gorukmez et al., 2022; Al Shamsi et al., 2025). Hence, no clear regional preference of the leukodystrophy can be associated with the patients described to date.

Symptoms of upper motor neuron degeneration were identified in 11 patients, with lower limb spasticity reported in nine cases and additional upper limb spasticity in five (Abu-Safieh et al., 2013; Ferdinandusse et al., 2017; Bartlett et al., 2021; Hasturk et al., 2024; Rudaks et al., 2024; Al Shamsi et al., 2025; Supplementary Table 1). Furthermore, a positive Babinski sign—indicative of corticospinal tract damage (Ambesh et al., 2017)—was observed in two patients (Hasturk et al., 2024; Al Shamsi et al., 2025). MRI findings support these clinical observations, revealing demyelination of the posterior crus of the internal capsule, which houses the corticospinal tracts, in five patients (Gorukmez et al., 2022; Hasturk et al., 2024; Al Shamsi et al., 2025). Additionally, spinal cord atrophy was noted in two patients (Bartlett et al., 2021; Rudaks et al., 2024), further indicating involvement of descending motor pathways.

In addition to motor fiber tracts, afferent sensory pathways also appear to be affected in some individuals (Supplementary Table 1). Hypesthesia was reported in two patients, but symptoms did not emerge until after the age of 15, significantly later than the onset of motor abnormalities (Rudaks et al., 2024; Al Shamsi et al., 2025). Nerve conduction studies (NCS) in these patients revealed pathological findings: the patient described by Rudaks et al. (2024) exhibited increased F-wave amplitudes and absent tibial somatosensory evoked potentials, while the patient reported by Al Shamsi et al. (2025) was diagnosed with peripheral motor neuropathy. Additionally, MRI scans in three patients revealed a leukodystrophy in the lemniscus medialis—an important fiber tract in the brain stem responsible for transmitting epicritic sensation and conscious proprioception (Ferdinandusse et al., 2017; Al Shamsi et al., 2025).

In summary, the widespread leukodystrophy and degeneration of both motor and sensory CNS tracts suggest that demyelination may originate at various, possibly random, locations within the CNS. However, it is notable that sensory symptoms were less common and had a later onset than motor deficits. Larger patient cohorts will be essential to identify regions of heightened vulnerability, with the corticospinal motor tracts appearing to be particularly susceptible in most currently described RDLKD cases.

#### 2.2.3 Gray matter alterations

Notably, in addition to white matter abnormalities in the CNS, MRI signal changes were also detected in motor function-related gray matter structures, including the basal ganglia (three patients; Rudaks et al., 2024; Al Shamsi et al., 2025) and the red nucleus (two patients; Al Shamsi et al., 2025). These findings suggest that RDLKD pathology is not confined to leukodystrophy but may also involve gray matter degeneration. However, cerebral atrophy—with dilatation of the sulci and ventricles on MRI scans—was observed in only two patients (Bartlett et al., 2021; Rudaks et al., 2024; Supplementary Table 1), indicating that widespread grey matter degeneration is potentially relatively rare.

#### 2.2.4 Cognitive impairments

Beyond motor and sensory deficits, cognitive impairments were reported in six patients (Ferdinandusse et al., 2017; Bartlett et al., 2021; Rudaks et al., 2024; Al Shamsi et al., 2025), and delayed language development in five (Ferdinandusse et al., 2017; Bartlett et al., 2021; Gorukmez et al., 2022; Hasturk et al., 2024; Al Shamsi et al., 2025; Supplementary Table 1). The cognitive symptoms ranged from mild learning difficulties (Al Shamsi et al., 2025) to severe deficits in speech and comprehension (Bartlett et al., 2021). The cognitive impairments typically emerge in childhood and appear to worsen during adolescence (Rudaks et al., 2024). Additionally, affective instability was reported in one patient (Rudaks et al., 2024), suggesting a possible neuropsychiatric component to RDLKD—a feature that remains largely unexplored in peroxisomal disorders. Interestingly, while one patient showed no cognitive impairment despite prolonged disease progression (Al Shamsi et al., 2025), febrile convulsions were reported in two others already during early childhood (Gorukmez et al., 2022), highlighting the broad phenotypic variability/severity of the disorder.

### 2.3 Other symptoms

Several anatomical abnormalities have been reported in RDLKD patients. Microcephaly was observed in six individuals (Ferdinandusse et al., 2017; Al Shamsi et al., 2025), while four patients were significantly underweight (Al Shamsi et al., 2025). Additionally, one patient was born with a cleft palate (Ferdinandusse et al., 2017; Supplementary Table 1). These findings suggest that the disease may manifest during early cranial development, potentially contributing to broader systemic dysfunction. However, based on the currently available patient data, it remains unclear whether the disorder involves defects in neuronal precursor proliferation or in the migration of neuronal/neural crest cells—processes that could underlie the development of microcephaly and craniofacial abnormalities.

Interestingly, one RDLKD patient exhibited endocrine ovarian insufficiency, reduced estradiol levels and elevated concentrations of luteinizing hormone and follicle-stimulating hormone in the blood (Rudaks et al., 2024; Supplementary Table 1). Adrenal gland dystrophy is a hallmark of X-linked adrenoleukodystrophy (X-ALD), implying a role of peroxisomes in steroidogenesis (Magalhaes and Magalhaes, 1997). Despite this, the role of peroxisomes in ovarian function remains poorly understood, and studies of the female reproductive system in relevant knockout mouse models are limited (Wang et al., 2022). Nonetheless, disruption of ovarian steroid metabolism could plausibly fall within the spectrum of peroxisomal dysfunction. This possibility warrants further investigation, particularly as advanced diagnostic tools increasingly facilitate the identification of patients with milder phenotypes.

In summary, ACBD5 deficiency (RDLKD) presents a complex and variable clinical phenotype, reflecting a broad spectrum of disease severity (Supplementary Table 1). Nonetheless, vision loss, cerebellar ataxia and nystagmus, as well as leukodystrophy emerge as consistent and early hallmarks in nearly all reported cases. Although infants often exhibit early motor dysfunction and cognitive deficits, disease progression appears relatively slow, with several patients surviving into midlife.

Biochemically (see section “2.4 Biochemical and cellular alterations in ACBD5-deficient patients”) and pathologically, RDLKD shares certain features with X-ALD, the most common peroxisomal disorder. Both conditions involve leukodystrophy; however, key differences help distinguish them. In X-ALD, white matter lesions typically extend from parieto-occipital to frontal regions (Thakkar et al., 2024), while in RDLKD, current MRI data do not suggest a consistent regional pattern. Notably, cerebellar involvement is prominent in RDLKD but not in X-ALD.

Visual impairment is another shared symptom, though with distinct underlying mechanisms. In X-ALD, optic nerve degeneration and inflammation are primary drivers, whereas in RDLKD, retinal degeneration—including loss of RPE—is the principal cause. Moreover, in X-ALD, retinal pathology typically manifests only in the severe cerebral form of the disease and is rarely observed in the milder adrenomyelopathy (Bianchi-Marzoli et al., 2021). In contrast, retinal dystrophy is one of the early clinical features in RDLKD patients and occurs, as also reported for less severe cases of the Zellweger spectrum (Karuntu et al., 2024), even in those with only mild motor or cognitive impairments (Al Shamsi et al., 2025).

The differences in retinal and cerebellar pathology between X-ALD and RDLKD may stem from distinct lipid metabolic alterations. In X-ALD, specifically saturated and monounsaturated VLCFAs accumulate in phospholipids. However, fibroblast data from RDLKD patients indicate a broader lipid profile that includes an accumulation of VLC-PUFAs. These are especially important for the normal function of the retina and cerebellum (Yeboah et al., 2021), which may account for the more pronounced pathologies observed in these tissues in RDLKD.

In addition, altered plasmalogen metabolism may also contribute to the tissue-specific manifestations of RDLKD. Nevertheless, due to the limited and partially conflicting data available (Herzog et al., 2018; Yagita et al., 2017), studies involving larger patient cohorts are needed to clarify the potential role of impaired plasmalogen biosynthesis in disease progression.

With respect to its phenotype, RDLKD bears partial resemblance to Refsum disease, which also features retinitis pigmentosa and cerebellar ataxia (Ruether et al., 2010). However, unlike Refsum disease, which stems from impaired branched-chain fatty acid metabolism, ACBD5-deficient patients typically have normal phytanoyl-CoA plasma levels.

As more patients are identified, the defining clinical features of RDLKD will become clearer, improving its distinction from other inherited peroxisomal disorders.

### 2.4 Biochemical and cellular alterations in ACBD5-deficient patients

Alterations in peroxisomal metabolism can be determined through the measurement of biomarkers such as VLCFA in plasma, or activity measurements of peroxisomal enzymes in patient skin fibroblasts (Wanders et al., 2017). Of the 11 known ACBD5 deficient patients, most exhibit impaired peroxisomal β-oxidation of VLCFA, reflected by elevated plasma levels of hexacosanoic acid (C26:0) (Table 1 and Supplementary Table 1). Out of nine patients measured for C26 plasma concentration, six displayed elevated levels while three patients were normal. Conversely, C24 plasma concentrations show variable changes, with two patients having elevated C24, two decreased, and two normal. The same is observed for C22 with one patient having elevated C22, two decreased, and two normal. To reveal if elevated VLCFA levels are a characteristic feature of ACBD5-deficiency, it may be required to determine C26 and VLCFA oxidation in patient fibroblast cultures (see below). ACBD5-deficient patients typically exhibit a 1.2-fold elevation of VLCFA over healthy individuals, while X-ALD patients exhibit an average of 4.5-fold elevation (Moser et al., 1999).

**TABLE 1:** Biochemical data of plasma samples from ACBD5-deficient patients.

Patient	Shamsi 1	Shamsi 3	Shamsi 4	Ferdinandusse	Yagita	Bartlett	Gorukmez 1	Gorukmez 2	Hasturk
Sex, age at report	M, 10	F, 7	F, 10	F, 9	Unknown	F, 36	F, 9	F, 5	M, 6
C26:0 (μmol/l)	1.36 (0.23 ± 0.09)	1.71 (0.00-1.08)	1.24 (0.00-1.08)	0.43 (0.05-0.41)	0.65 (0.45-1.32)	1.02 (0.31-0.81)	0.71 (< 0.92)	1.26 (< 0.92)	0.8 (< 1.3)
C24:0 (μmol/l)	-	84.25 (18.99-72.54)	-	-	20 (33-84)	32.74 (35.6–101.6)	111.93 (< 80)	76.15 (< 80)	12 (< 91.4)
C22:0 (μmol/l)	-	-	-	-	20.5 (40-119)	31.5 (42.9–112.7)	129.65 (< 105)	88.37 (< 105)	10 (< 96.3)
C26:0/C22:0	0.27 (0.01-0.004)	Normal	-	0.025 (0.002–0.018)	0.032 (< 0.028)	0.03 (0.0049-0.0118)	0.005 (0.006-0.014)	0.01 (0.006-0.014)	0.08 (< 0.023)
C26:0/C24:0	-	Normal	-	-	-	-	-	-	-
C24:0/C22:0	-	-	1.288 (0-1.158)	1.29 (0.64-1.04)	0.976 (< 1.2)	1.04 (0.726-0.988)	0.86 (0.51-1.19)	0.86 (0.51-1.19)	1.2 (< 1.39)
C26-Carnitine (μmol/l)	-	-	-	0.099 (0.014-0.077)	-	-	-	-	-
C26:0 LysoPC (μmol/l)	-	-	-	0.166 (29-72)	-	-	-	-	-
Phytanic acid (μmol/l)	-	Normal	-	-	-	0.33 (0.58-2.54)	0.23 (0.04-11.5)	0.23 (0.04-11.5)	-
Pristanic acid (μmol/l)	-	-	-	-	-	0.12 (0.11-0.41)	0.62 (< 3.4)	0.59 (< 3.4)	-
Prist/Phyt ratio	-	-	-	-	-	0.35 (0.093-0.254)	-	-	-

Reference data provided from each report listed in brackets. Elevated (blue) and reduced (purple) compared to control.

#### 2.4.1 Investigation of patient fibroblasts

To date, three studies have explored the biochemical data of patient derived skin fibroblasts (Ferdinandusse et al., 2017; Yagita et al., 2017; Herzog et al., 2018). As expected, ACBD5-deficient patient fibroblast cultures demonstrate a clear impairment in peroxisomal β-oxidation of VLCFAs, leading to their accumulation (Table 2). C26 elevation remains consistent between patient plasma and cell culture. This is in keeping with the C24/C22 ratio, however, there are discrepancies with the C26/C22 ratio, where plasma of most patients presents an elevated ratio while the patient fibroblasts remain normal (Ferdinandusse et al., 2017). Patient fibroblasts display a reduced C22 and C24 level with an elevated C26 concentration which is indicative of impaired peroxisomal β-oxidation of C26, while mitochondrial β-oxidation might be increased potentially via peroxisome proliferator activated receptor (PPAR) α activation. Similar observations have been made in ACBD5 knock out cells (Yagita et al., 2017; Costello et al., 2023).

**TABLE 2:** Biochemical analysis of ACBD5-deficient patient fibroblasts.

(A)						
VLCFA		VLCFA loading tests				
			Patient	X-ALD	Control 1	Control 2
C22:0 (μmol/l)	3.01/4.4 (3.84–10.20)	D3-C28:0 (μmol/g)	0.22	0.13	n.d.	n.d.
C24:0 (μmol/l)	7.65/9.23 (7.76–17.66)	D3-C26:0 (μmol/g)	1.53	1.86	0.67	0.24
C26:0 (μmol/l)	1.07/1.14 (0.18–0.38)	D3-C24:0 (μmol/g)	12.87	19.93	12.75	11.02
C26:0/C22:0 (μmol/l)	0.35/0.26 (0.03–0.07)	C26:0 (μmol/g)	0.26	0.45	0.01	0.02
C24:0/C22:0 (μmol/l)	2.54/2.10 (1.55–2.30)	C26:0/C22:0	0.15	0.17	0.01	0.01
C26:0 LysoPC (μmol/l)	32 (2–14)	C24:0/C22:0	2.27	2.25	2.19	1.97
**(B)**						
**Peroxisome functional analysis**				**ACBD5 rescue**		
**Fatty acid oxidation activity**	**[pmol/(h.mg)]**		**Mock**		**ACBD5 rescue**	
Phytanic acid α-oxidation	31 (28–95)	D3-C28:0 (μmol/g)	0.17 ± 0.01		0.05 ± 0.01*	
Pristanic acid β-oxidation	1086 (748–975)	D3-C26:0 (μmol/g)	2.20 ± 0.22		1.13 ± 0.13*	
C26:0 β-oxidation	437 (1,273–1,431)	D3-C24:0 (μmol/g)	15.30 ± 1.51		12.52 ± 0.48	
Activity of peroxisomal enzymes	[nmol/(2h.mg)]	C26:0 (μmol/g)	0.35 ± 0.02		0.14 ± 0.06*	
DHAPAT	9.8 (5.4–10.6)	C26:0/C22:0	0.31 ± 0.13		0.15 ± 0.05	
ACOX1	285 (74–206)	C24:0/C22:0	2.57 ± 0.96		2.60 ± 0.21	
DBP hydratase	143 (115–600)	C26:0 lysoPC (μmol/g)	29		18	
DBP dehydrogenase	54 (25–300)					
SCPX	20 (10–39)					

**(A)** VLCFA analysis in ACBD5-deficient patient skin fibroblasts. **(B)** Functional analysis of peroxisomes from ACBD5-deficient patient skin fibroblasts and peroxisomal β-oxidation rescue through introduction of functional ACBD5. *Statistically significant rescue (*P* < 0.005). Elevated (blue) and reduced (purple) compared to control [adapted from Ferdinandusse et al. (2017) with permission from the publisher].

The functional complementation of patient cells with wild-type ACBD5, which restored normal VLCFA metabolism, confirms the causative role of ACBD5 defects in compromised VLCFA handling (Table 2). No abnormalities in phytanic acid α-oxidation were detected, reinforcing the notion that ACBD5 is specifically required for β-oxidation of VLCFA (Ferdinandusse et al., 2017; Yagita et al., 2017). The oxidation of the breakdown product of phytanic acid, pristanic acid, was only marginally raised, indicating functionality of the peroxisomal β-oxidation pathway itself. Furthermore, all peroxisomal enzymes except for ACOX1 were found to be in normal ranges (Table 2). ACOX1, the key enzyme in peroxisomal β-oxidation (Figure 1), may be raised as a response to the over-abundance of distinct VLCFAs activating PPARs, suggesting that peroxisomes are functional but the lack of ACBD5 impedes peroxisomal VLCFA availability for β-oxidation.

Notably, despite a role for ACBD5 in PO-ER contact site formation and phospholipid transfer for membrane expansion, total peroxisomal number is not affected (Yagita et al., 2017). Provided that peroxisome turnover is not decreased, the biogenesis of peroxisomes appears not to be significantly reduced by a lack of ACBD5, nor is the import of peroxisomal proteins disrupted (Yagita et al., 2017). The presence of other PO-ER tether proteins such as ACBD4 may partially compensate in peroxisome biogenesis (Costello et al., 2023). Alternatively, *de novo* biogenesis from the ER might compensate for a reduced membrane extension capacity of the growth and division pathway.

It should be noted that, in contrast to its yeast ortholog Atg37 (Nazarko, 2014; Nazarko et al., 2014), the potential impact of human ACBD5 on peroxisome degradation/pexophagy is still unresolved. While RNAi-mediated knockdown of ACBD5 in HeLa cells reduced peroxisomal autophagy rates (Nazarko et al., 2014), knockout of ACBD5 in HeLa and other mammalian cells did not affect pexophagy (Ferdinandusse et al., 2017; Kors et al., 2024). It should, however, be mentioned that a recent study reported a correlation between kidney fibrosis and a reduction in pexophagy, which was associated with downregulation of ACBD5 (Kim et al., 2025).

Investigation of ACBD4/5 orthologs in other species such as the filamentous fungus *Ustilago maydis* (Um) revealed that a loss of Um_ACBD4/5-like protein causes an increase in lipid droplet numbers, and a decline in mitochondrial membrane potential, implying altered lipid homeostasis, which impacts on several organelles. If other organelles are also affected in ACBD5-deficient patient cells needs to be demonstrated.

It is possible that ACBD5 is involved in the formation of other membrane contacts besides its role in PO-ER tethering. In addition, ACBD5 may be involved in signaling processes via fatty acids, or at membrane contacts. Alterations of inter-organelle communication/signaling and/or the impact of altered lipid metabolism on other organelles could contribute to the diverse symptoms of RDLKD.

Lipidomic analysis of ACBD5 patient cells revealed an increase in VLCFA accumulation within phospholipids, reflecting similar results from ACOX1-, DBP-, and X-ALD-deficient fibroblasts (Herzog et al., 2018), along with a decrease in ether-phospholipids. By contrast, Fujiki and coworkers only observed the accumulation of VLCFA in fibroblasts from another ACBD5-deficient patient, while ether lipids and DHA were not altered (Yagita et al., 2017). The consistent VLCFA accumulation may be a consequence of impaired palmitoyl-CoA synthesis, which is primarily performed in peroxisomes from C26:0-CoA β-oxidation (Herzog et al., 2018) but could further be related to reduced membrane contacts between peroxisomes and the ER, both of which cooperate in ether lipid synthesis. This phospholipid deficiency extends to phosphatidylethanolamine-ether and phosphatidylcholine-ether phospholipids, and therefore pointing to a general phospholipid biosynthesis defect, including a general decrease in phosphatidylethanolamine-plasmalogens (Hua et al., 2017; Herzog et al., 2018). It should be noted that, however, different patient derived cultures from separate research groups identified normal phosphatidylethanolamine-plasmalogen levels (Yagita et al., 2017). In contrast to ABCD1-deficient fibroblasts, where only VLC-PC species containing unsaturated, monounsaturated, and disaturated VLCFAs accumulate, in ACBD5-deficient fibroblasts an accumulation of broad VLC-PC species including VLC-PUFAs was observed (Yagita et al., 2017). Thus, ACBD5 appears to bind and transfer VLC-CoA without a strong preference for a specific ABCD transporter, but likely cooperates with ABCD1, ABCD2, and possibly ABCD3 to facilitate the import of a broad spectrum of fatty acids destined for peroxisomal degradation.

## 3 Mouse models for the ACBD5-deficiency

### 3.1 Generation of *Acbd5*-deficient mouse models

To date, insights into the cellular and metabolic consequences of ACBD5 deficiency in humans are derived exclusively from fibroblast cultures and immortalized cell lines, where ACBD5 was inactivated using CRISPR-Cas9 technology. As a result, knowledge of ACBD5’s role in terminally differentiated cells—such as neurons—and in complex tissues remains limited. To address this gap, two ACBD5-deficient mouse models have been developed. In the *Acbd5*^*tmn*1*a*^ model, transcription of the *Acbd5* gene is disrupted by a neomycin resistance cassette inserted between exons 2 and 3 (Skarnes et al., 2011; Darwisch et al., 2020). In the *Acbd5*^*Gly*357*^ model, a premature stop codon was introduced into exon 9 of the *Acbd5* gene via CRISPR-Cas9, resulting in complete loss of ACBD5 protein expression (Granadeiro et al., 2024). Both *Acbd5*^–/–^ mouse strains display approximately twofold increases in hexacosanoic acid (C26:0) levels in tissues and plasma, mirroring biochemical abnormalities observed in ACBD5-deficient patients (Darwisch et al., 2020; Granadeiro et al., 2024). These models thus validate the impaired peroxisomal β-oxidation capacity previously demonstrated in patient-derived fibroblasts (Ferdinandusse et al., 2017; Yagita et al., 2017).

### 3.2 Neurological and retinal pathology

Both ACBD5-deficient mouse models exhibit prominent neurological phenotypes, including hindlimb-clasping reflex loss and ataxia, closely reflecting the locomotor dysfunction seen in patients. Consistent with these motor deficits, both *Acbd5*^–/–^ models show cerebellar degeneration marked by Purkinje cell loss, pronounced axonal swellings, and demyelination of cerebellar white matter tracts (Darwisch et al., 2020; Granadeiro et al., 2024), which correlates with the cerebellar symptoms typically observed in RDLKD patients. Brites and colleagues further reported myelin thinning in the spinal cord and the presence of giant axonal swellings accompanied by disorganized axonal filament proteins, highlighting structural disruptions likely contributing to motor impairment (Granadeiro et al., 2024). Moreover, their ACBD5-deficient mice developed a significant gliosis throughout the white and gray matter of the telencephaolon, which correlates with the brain degeneration observed in RDLKD patients. *In vitro*, *Acbd5*^–/–^ cortical neurons treated with tetracosanoic acid methyl ester (C24) displayed reduced actin dynamics, suggesting that lipidomic imbalances may impair cytoskeletal regulation—potentially underlying the neurological manifestations of ACBD5 deficiency.

Neuroinflammation, a hallmark of leukodystrophies, is also evident in both mouse models. Significant astrocyte and microglial activation was observed in the cerebellum, with additional neuroinflammatory changes identified in the telencephalon of *Acbd5*^*Gly*357*^ mice (Granadeiro et al., 2024). Like human patients, both mouse strains developed retinal dystrophy, characterized by photoreceptor cell loss and microglial activation (Darwisch et al., 2020; Granadeiro et al., 2024). However, a detailed characterization of the retinal pathology and identification of affected cell types in ACBD5-deficient mice remains pending.

### 3.3 Biochemical alterations

#### 3.3.1 VLCFAs and DHA

At the molecular level, impaired peroxisomal β-oxidation in the ACBD5-deficient mice leads to elevations of plasma VLCFAs and to significant, tissue-specific alterations in lipid composition, thus closely resembling the lipid alterations observed in plasma and fibroblasts from RDLKD patients (Darwisch et al., 2020; Granadeiro et al., 2024). As expected from elevated VLCFA levels, all analyzed tissues—liver, cerebellum, and spinal cord—show increased incorporation of VLCFAs into phosphatidylcholines. While fatty acid chains in these phospholipids typically do not exceed C36, nervous tissue phosphatidylcholines in *Acbd5*^–/–^ mice can reach up to C42. Additionally, fatty acids accumulating in membrane phospholipids in the CNS exhibit a higher degree of unsaturation compared to those in the liver (Darwisch et al., 2020). These organ-specific differences in chain length and number of double bonds likely result from the tissue-specific expression of fatty acid elongases and desaturases (Ferrero et al., 2025).

Fatty acid chain length is governed by a balance between elongation and degradation, a process thought to be regulated at ER–peroxisome contact sites (Wanders et al., 2018; Schrader et al., 2020). Disruption of these contact zones in ACBD5-deficient cells may lead to a sustained fatty acid elongation at the ER (Figure 3). This can result in an accumulation of ultra-long chain fatty acids in tissues expressing ELOVL4—the only elongase capable of extending fatty acids beyond C32. ELOVL4 is selectively expressed in the skin, testis, and nervous system (Yeboah et al., 2021), and its activity, along with that of CNS-specific desaturases, may partly explain the predominantly neurological phenotype in ACBD5-deficient mice and humans.

Originating from linolenic acid, the synthesis of DHA requires three consecutive rounds of elongation and desaturation at the ER followed by a single, final chain-shortening step by peroxisomal β-oxidation (Wanders et al., 2023). Disruption of ER-peroxisome contacts could therefore impair DHA synthesis in *Acbd5*^–/–^ cells (Figure 3). However, DHA levels were only slightly reduced in liver lysophosphatidylcholines, and no differences were detected in nervous tissue between *Acbd5*^–/–^ and wild-type mice (Darwisch et al., 2020; Granadeiro et al., 2024), suggesting compensatory mechanisms, possibly via upregulated DHA synthesis. Interestingly, *Acbd5*^*tmn*1*a*^ mice display significantly increased peroxisome numbers in hepatocytes, which may partially compensate for the reduction in peroxisomal β-oxidation capacities and supply (Darwisch et al., 2020). This compensation could help explain why the *Acbd5*^–/–^ mice, despite marked neuropathology, often survive beyond 1 year. In contrast, humans lack PPARα-mediated peroxisome proliferation, limiting such compensation and possibly resulting in a more severe clinical course. Data from patient fibroblasts do not indicate a significant reduction of DHA in phospholipids (Herzog et al., 2018; Yagita et al., 2017). Accordingly, DHA synthesis or dietary uptake appears sufficient to maintain adequate levels in membrane lipids.

In addition to the marked accumulation of VLCFAs in phosphatidylcholines and triglycerides in *Acbd5*^–/–^ mice, more subtle alterations are also observed in lipid species with shorter chain lengths and across various lipid classes. These complex lipid changes are particularly pronounced in the spinal cord of *Acbd5*^*Gly*357*^ mice (Granadeiro et al., 2024). Given that demyelination significantly impacts the long ascending and descending fiber tracts of the spinal cord, it remains unclear whether these profound changes in lipid composition reflect a broader disruption of lipid homeostasis in the spinal cord or are primarily driven by the reduction in specialized myelin membrane lipids.

#### 3.3.2 Ether lipids

Ether lipid synthesis requires the cooperation of peroxisomes and the ER (Wanders et al., 2023). While the initial steps required for the formation of the alkyl ether bond at the *sn1*-position occur in peroxisomes, the pathway is completed at the ER by coupling an acyl group at the *sn2*-position and addition of a PC or PE head group (Malheiro et al., 2015). Fibroblasts from ACBD5-deficient patients showed in part reduced levels of ether phospholipids, indicating that efficient ether lipid synthesis depends on the physical interaction between these two organelles (Herzog et al., 2018; Figure 3).

Lipidomic data from *Acbd5*^*tmn*1*a*^ mice reveal a moderate reduction of alkyl-PE and alkyl-PC in the cerebellum, but not in the liver. Notably, levels of alkyl-diacyl- and alkyl-acylglycerols are approximately doubled in the liver (Darwisch et al., 2020). This suggests that increased peroxisome numbers in the liver may overcompensate for reduced ether lipid synthesis, whereas such compensation does not appear to occur in the cerebellum. Interestingly, although orally administered alkylglycerols enter the bloodstream, they do not seem to cross the blood-brain barrier effectively (Dorninger et al., 2022). This may explain why *Acbd5*^–/–^ mice are unable to compensate for CNS ether lipid deficits through hepatic export of surplus alkylglycerols. It remains unclear whether the peroxisome proliferation observed in *Acbd5*^–/–^ mice is mediated by PPARα, which could limit the relevance of this compensatory response in human patients. Furthermore, data from additional tissues in the mouse models, as well as a larger number of patient samples, are needed to determine whether plasmalogen deficiency is a consistent feature of ACBD5 deficiency or a secondary, less critical factor in the disease pathology.

#### 3.3.3 Cholesterol

In response to the siRNA mediated knockdown of ACBD5 and/or VAP proteins in HeLa cells reductions in cellular cholesterol levels were reported (Hua et al., 2017). This observation might relate to the hypothesis of a peroxisome-localized presqualene segment of the cholesterol synthesis pathway in peroxisomes requiring the transfer of intermediates to the ER for pathway completion (Faust and Kovacs, 2014). However, the involvement of peroxisomes in mammalian cholesterol synthesis remains controversial (Hogenboom et al., 2003). Alternatively, peroxisomes were proposed to function as hubs for the transfer of cholesterol to the ER for degradation (Chu et al., 2015; Yamashita et al., 2023). In addition, dysregulation of peroxisomal β-oxidation in X-ALD models has been shown to impact cholesterol homeostasis (Buda et al., 2023). Hence, peroxisomal metabolism may play a role within a broader regulatory network governing cholesterol homeostasis (Charles et al., 2020). It is important to note, however, that no alterations in cholesterol levels have been reported in ACBD5-deficient patients to date. Further studies are therefore warranted to better assess the potential impact of ACBD5 deficiency on cholesterol homeostasis.

#### 3.3.4 Comparison with knockout mouse models of other peroxisomal proteins

Retinal dystrophy with leukodystrophy is characterized by relatively early onset and severe pathology, though it does not appear to be associated with high mortality (*see* above). In contrast, PEDs directly affecting β-oxidation (Figure 1)—such as ACOX1 and MFP2/BP deficiencies—present with neonatal onset and typically lead to death within the first few years of life (Huyghe et al., 2006; Ferdinandusse et al., 2007). Patients with X-ALD, caused by mutations in ABCD1, the transporter responsible for peroxisomal import of straight-chain VLCFAs, often develop the severe cerebral form of the disease between ages 3 and 10, with rapid progression and neuroinflammation (Engelen et al., 2012). A milder phenotype, adrenomyeloneuropathy, manifests later—typically in the third or fourth decade—patients do not develop a cerebral leukodystrophy.

Within this broad phenotypic spectrum, comparing pathology in *Acbd5*^–/–^ mice to other PED mouse models may help identify disease-specific features of RDLKD and clarify its underlying mechanisms. Like *Mfp2*^–/–^ mice, which have a complete block in VLCFA β-oxidation, *Acbd5*^–/–^ mice show cerebellar and retinal pathology accompanied by neuroinflammation. However, the onset in *Mfp2*^–/–^ mice occurs earlier—within the first weeks after birth—and with a profoundly more severe symptomatology (Verheijden et al., 2013). Lipidomic analyses of *Mfp2*^–/–^ retinas, which also exhibit early and severe degeneration, reveal significant accumulation of VLC-PUFAs in the CNS (Das et al., 2021). Notably, *Mfp2*^–/–^ mice also display reduced levels of DHA in membrane lipids, a feature not observed in *Acbd5*^–/–^ mice.

While *Acox1*^–/–^ mice present with a prominent liver pathology, information on the degeneration of the CNS is limited (Hashimoto et al., 1999). Recent studies have reported early-onset retinal degeneration, marked by photoreceptor loss and shortage of DHA-containing lipids as early as 1 month of age (Boeck et al., 2025). Importantly, dietary DHA supplementation significantly ameliorated disease progression in both *Mfp2*^–/–^ and *Acox1*^–/–^ models, suggesting that DHA depletion may be a key driver of CNS pathology (Swinkels et al., 2023; Boeck et al., 2025).

Very-long-chain fatty acid import into peroxisomes is mediated by the ABC transporters ABCD1 and ABCD2, which have overlapping but distinct substrate preferences (Tawbeh et al., 2021) (Figure 1). Both transporters can import saturated and monounsaturated VLCFAs, but only ABCD2 appears to have substrate specificity for VLC-PUFAs, including DHA. These molecular differences are also reflected *in vivo*: *Acbd1*^–/–^ mice exhibit a roughly sixfold increase in C26:0 levels in the spinal cord—approximately three times higher than in *Acbd5*^–/–^ mice—whereas *Acbd2*^–/–^ mice show no such accumulation (Pujol et al., 2004; Granadeiro et al., 2024).

Interestingly, *Acbd1*^–/–^ mice, despite high VLCFA accumulation, do not develop cerebral disease and instead model the milder peripheral adrenomyelopathy, with late-onset symptoms appearing around 20 months of age, whereas *Acbd2*^–/–^ mice did not develop symptoms (Pujol et al., 2004). In contrast, onset of locomotor symptoms was observed significantly earlier in *Abcd2*^–/–^ than in *Abcd1*^–/–^ mice. Moreover, *Acbd2*^–/–^ mice develop signs of ataxia corresponding to a significant decline in cerebellar Purkinje neurons at the age of 20 months, suggesting that the more severe locomotor phenotype is associated with the degeneration of central motor centers (Ferrer et al., 2005).

Phenotypically, *Acbd5*^–/–^ mice more closely resemble the *Abcd2^–/–^* model, though they exhibit earlier onset and more rapid progression. Since *Acbd1*^–/–^ mice accumulate the highest levels of straight-chain VLCFAs without developing CNS pathology, these lipid species likely play a minor role in *Acbd5*^–/–^ disease progression. Instead, the accumulation of VLC-PUFAs may be more relevant. Additionally, other factors—such as disturbances in plasmalogen levels or non-lipid metabolites associated with ACBD5’s ER-tethering role—may contribute to pathogenesis. In this context, the *Acbd5*^*Gly*357*^ and *Acbd5*^*tmn*1a^ mouse models are valuable tools for identifying tissue- or cell-specific metabolic and proteomic alterations that may underlie the selective degeneration observed in specific organs.

In summary, considering relative age, the phenotype in *Acbd5^–/–^* mice appears to develop somewhat more slowly than in humans, with disease onset not occurring before early adulthood. Nevertheless, the key biochemical abnormalities, affected tissues, and the progressive nature of the phenotype—which ultimately leads to considerable severity—show substantial similarities between mice and humans. However, species-specific differences in liver metabolism, retinal cell morphology, and CNS complexity limit the direct translatability of findings from mice to patients. Despite these limitations, *Acbd5*-deficient mouse models remain valuable tools for elucidating the fundamental mechanisms underlying RDLKD pathogenesis.

## 4 Conclusion and outlook

### 4.1 Toward an understanding of ACBD5 function in health and disease

Acyl-CoA Binding Domain-Containing Protein 5 deficiency is a newly recognized, severe peroxisomal disorder characterized by a complex and variable range of symptoms (Supplementary Table 1 and Figure 3). Leukodystrophy, motor impairments combined with early ophthalmological alterations during childhood appear to be key features of the disease and combined with elevated VLCFA plasma levels, should prompt consideration of RDLKD. Notably, early childhood retinal dystrophy paralleled by a nystagmus might be considered as a sign for further analyses toward mutations in ACBD5. However, the low number of diagnosed cases is currently limiting detailed conclusions on characteristic symptoms, which may aid a precise diagnosis and discrimination from other peroxisomal disorders.

Like other peroxisomal single-enzyme deficiencies that do not entirely block a metabolic pathway—such as X-ALD—ACBD5 deficiency presents with a broad spectrum of disease severity. This variability may reflect individual responses to metabolic alterations influenced by the patient’s genetic background. Additionally, the dual role of ACBD5 may contribute to the disorder’s heterogeneity. ACBD5 functions both as an acyl-CoA binding protein involved in peroxisomal fatty acid import and as a mediator of ER-peroxisome MCSs. Loss of ACBD5 can therefore disrupt multiple pathways (e.g., ether lipid-, bile acid, or DHA synthesis; balancing the equilibrium between VLCFA elongation and breakdown) that rely on the interplay between these organelles (Figure 3; Wanders et al., 2018). Moreover, as seen in X-ALD, variations in CNS inflammation may play a significant role in the heterogeneity of disease severity and warrant further investigation in the expanding RDLKD patient cohort.

The accumulation of VLCFAs, their subsequent incorporation into phospholipids, and altered lipid spectrum appears to correlate well with the neurological abnormalities in ACBD5 deficiency. In this context, the early retinal and cerebellar symptoms observed in RDLKD patients align with cytological abnormalities seen in *Acbd5*-deficient mouse models. Notably, these two tissues exhibit the highest expression levels of ELOVL4, the elongase responsible for synthesizing VLCFAs (Yeboah et al., 2021; Sherry et al., 2017). Given ELOVL4’s essential role in VLC-PUFA synthesis, the marked accumulation of these fatty acids in cerebellar phospholipids of *Acbd5*-deficient mice may suggest their involvement in RDLKD pathogenesis. Further lipidomic analyses of patient-derived fibroblasts, as well as specific tissues from *Acbd5*^–^ mouse models, are needed to determine whether disruptions in ether lipids, DHA, cholesterol, or bile acid metabolism also contribute to the development of the pathogenesis in distinct tissues.

Such alterations in membrane lipid compositions could also impact on cellular signaling functions and/or generate cellular stress, which could impact on disease progression. The impact of a loss of the ACBD5 tethering function and the metabolic cooperation/communication with the ER on the disease pathology is currently not well understood. In addition to its peroxisome-ER tethering function, ACBD5 may also mediate contacts with other organelles involved in lipid metabolism, such as mitochondria or lipid droplets. Furthermore, ACBD5 may have a direct role in fatty acid/lipid signaling. Its coiled-coil domain mediates homo-oligomerization, but also hetero-oligomerization with ACBD4 (Costello et al., 2023), suggesting a more complex role at the peroxisomal membrane. Further studies are therefore required to identify interaction partners of ACBD5 and to determine the key metabolites and pathways affected by ACBD5 loss, as this will be critical for developing targeted pharmacological therapies.

### 4.2 Treatment options and challenges for future therapies

Currently, there is no cure for ACBD5 deficiency, and treatment remains largely supportive. However, several therapeutic strategies are under investigation.

As ACBD4 expression can compensate for the loss of ACBD5 in peroxisome-ER tethering and VLCFA β-oxidation in cell culture (Costello et al., 2023), pharmacological upregulation of ACBD4 expression may be an approach to overcome loss of ACBD5. However, ACBD4 also appears to differ functionally from ACBD5 and may have regulatory functions in peroxisomal lipid metabolism (Costello et al., 2023). Furthermore, it is not ubiquitously expressed in all tissues and also transcribed into splice variants lacking the TMD-coding segment. Pharmacological approaches aimed at correcting metabolic imbalances and dietary interventions to manage VLCFA accumulation as used in other peroxisomal disorders such as X-ALD may be beneficial but are largely unexplored.

Gene therapy offers a promising avenue; preclinical studies using adeno-associated virus (AAV)-mediated delivery of a functional ACBD5 gene have shown significant improvement in disease progression in the *Acbd5*^Gly357*^ mouse model (Granadeiro et al., 2024). However, to fully halt disease pathogenesis, efficient transduction of neural cells may be required, necessitating viral vectors capable of efficiently crossing the blood-brain barrier.

For cerebral ALD, hematopoietic cell transplantation has been successful, however, to be effective should be performed before or at the beginning of the development of pathologic symptoms (Wolf et al., 2025). One of the reported RDLKD patients received an autologous intrathecal hematopoietic stem cell transplantation at the age of 29, which lead to a transient, partial improvement of vision, speech and locomotor abilities (Bartlett et al., 2021). Considered that the stem cell therapy was administered considerably after the onset of the disease pathology (at the age of 3), a treatment of RDLKD patients before or at the beginning of the development of symptoms might more effectively ameliorate disease progression. Furthermore, an autologous hematopoietic stem cell transplantation after *ex vivo* lentiviral gene therapy has been developed (Eichler et al., 2017). Although its relevance to ACBD5 deficiency remains to be established, current advances in the treatment options for leukodystrophies may also benefit the treatment of ACBD5 patients (Metovic et al., 2024; Wolf et al., 2025).

Given that RDLKD is a neurodegenerative disorder, early therapeutic intervention—ideally in early childhood—is essential to prevent irreversible damage. However, standard neonatal screening may miss affected individuals, as fatty acid profiles can appear normal in some ACBD5-deficient patients. Therefore, genetic screening and advanced metabolomics will be crucial for reliably identifying RDLKD among patients presenting with unexplained retinal dystrophy or leukodystrophy. Such efforts will not only improve diagnostic accuracy but also enhance our understanding of the RDLKD phenotype spectrum and reveal its true prevalence, which is likely underestimated due to current diagnostic limitations.
