# Supplementary material for: The neurological pathology of peroxisomal ACBD5 deficiency – lessons from patients and mouse models
Source: Front Mol Neurosci. 2025 Jul 2;18:1602343. doi: 10.3389/fnmol.2025.1602343 (PMC12263615; doi:10.3389/fnmol.2025.1602343)
Supplement: Supplementary file 1 [file Table_1.docx]

Supplementary Material

**Supplementary Table S1** Overview of symptoms of ACBD5 patients

|  | **Gorukmez1** | **Gorukmez2** | **Rudaks** | **Bartlett** | **Ferdinand.** | **Hasturk** | **Al-Shamsi1** | **Al-Shamsi2** | **Al-Shamsi3** | **Al-Shamsi4** | **Al-Shamsi5** | **Abu-Safieh** | **Pappat.1** | | **Pappat.2** | |  |
| --- | --- | --- | --- | --- | --- | --- | --- | --- | --- | --- | --- | --- | --- | --- | --- | --- | --- |
| **General background** | | | | | | | | | | | | | |  | |  | |
| **Mutation** | c.1297C>T, p.Arg433*,  stop codon in exon 10 | c.1297C>T, p.Arg433*,  stop codon in exon 10 | c.979G > T, p.Gly327* c.399del, p.Ile134Leufs*6, 2 stop codons | c.1467G>A, p.(Trp489*) stop codon in exon12 | 936+1075_c.936+  deletion exons 7-8,  p.Asp208  Valfs*30 | c.936+2T>G novel intronic splice site between exon 7 - 8 | exon deletion  7-8 | exon deletion  7-8 | exon deletion  7-8 | exon deletion 4 | exon deletion 4 | c.1205+1G-A  p.Gly402  Aspfs*5,  stop codon in exon 10 | c.431G>A, p.Gly144Asp  amino acid exchange | | c.431G>A, p.Gly144Asp  amino acid exchange | |  |
| **Sex, age at report** | f, 9 y | f, 5 y | f, 30 y | f, 36 y | f, 9 y | m, 6 y | m, 10 y | m, 4 y | w, 7 y | w, 10 y | m, 30 y | n.a. (3) | m, 48 y | | f, 18 y | |  |
| **Fatty acid serum concentrations** | | | | | | | | | | | | | | | | | |
| C22:0 | / | + | / | − | / | / | / | n.a. | n.a. | n.a. | n.a. | n.a. | n.a. | | n.a. | |  |
| C24:0 | / | + | / | − | / | / | / | n.a. | + | n.a. | n.a. | n.a. | n.a. | | n.a. | |  |
| C26:0 | + | / | / | + | + | / | + | n.a. | + | + | n.a. | + | n.a. | | n.a. | |  |
| C24:C22 ratio | / | / | / | + | + | / | / | n.a. | n.a. | + | n.a. | n.a. | n.a. | | n.a. | |  |
| C26:C22 ratio | / | / | / | + | / | + | + | n.a. | n.a. | n.a. | n.a. | + | n.a. | | n.a. | |  |
| Phytanic acid | n.a. | n.a. | n.a. | − | / | / | + | n.a. | n.a. | n.a. | n.a. | n.a. | n.a. | | n.a. | |  |
| Pristanic acid | n.a. | n.a. | n.a. | (−) | / | / | + | n.a. | n.a. | n.a. | n.a. | n.a. | n.a. | | n.a. | |  |
| **Symptoms [onset in months]** | | | | | | | | | | | | | | | | | |
| First symptom onset | 12 | 12 | 2 | 1 | 7 | 2 | 3 | 3 | 5 | 3 | <6 | n.a. | n.a. | | n.a. | |  |
| **Opththalmological** |  |  |  |  |  |  |  |  |  |  |  |  |  | |  | |  |
| Photosensitivity/Photphobia | 12 | 12 | 2 | n.a. | n.a. | n.a. | 24 | 5 | 5 | 3 | <6 | n.a. | n.a. | | n.a. | |  |
| Visus loss | 36 | 36 | 2 | 36 | 7 | 60 | 24 | 48 | 18 | 16 | <6 | n.a. | 576 | | × | |  |
| Dyschromatopsia | n.a. | n.a. | 18 | n.a. | n.a. | n.a. | n.a. | n.a. | n.a. | n.a. | n.a. | n.a. | 576 | | × | |  |
| Nyctalopia | n.a. | n.a. | n.a. | n.a. | n.a. | n.a. | n.a. | n.a. | n.a. | n.a. | n.a. | n.a. | 576 | | × | |  |
| Myopia | n.a. | n.a. | n.a. | n.a. | n.a. | n.a. | 24 | × | 18 | n.a. | <6 | n.a. | n.a. | | × | |  |
| Astigmatism | n.a. | n.a. | n.a. | n.a. | n.a. | n.a. | 24 | 48 | 18 | 16 | × | n.a. | n.a. | | n.a. | |  |
| Dysfunct. eye motility |  |  | 180 | n.a. | 7 | 72 | n.a. | n.a. | n.a. | - | n.a. | n.a. | n.a. | | n.a. | |  |
| **Neurological** | | | | | | | | | | | | | | | | | |
| Nystagmus | 12 | 12 | 2 | 1 | 48 | 2 | 3 | 5 | 5 | 3 | <6 | n.a. | n.a. | | n.a. | |  |
| Lower body spasticity | n.a. | n.a. | 24 | 36 | 48 | 72 | 14 | × | 11 | 36 | 180 | + | n.a. | | n.a. | |  |
| Upper body spasticity | n.a. | n.a. | 72 | 180 | 48 | n.a. | 14 | n.a. | 84 | n.a. | × | n.a. | n.a. | | n.a. | |  |
| Hypotonia | n.a. | n.a. | 24 | 36 | 48 | 72 | 120 | 48 | 84 | n.a. | n.a. | n.a. | n.a. | | n.a. | |  |
| Psychomotor regression | 24 | 36 | 24 | 132 | 14 | 48 | 120 | 36 | 84 | 24 | 180 | + | n.a. | | n.a. | |  |
| Inability to walk | 36 | 48 | 72 | 156 | 108 | 0 | 120 | 36 | 84 | 36 | 360 | n.a. | n.a. | | n.a. | |  |
| Ataxia | n.a. | n.a. | 24 | n.a. | 48 | n.a. | n.a. | n.a. | n.a. | n.a. | 204 | n.a. | n.a. | | n.a. | |  |
| Intention tremor | 48 | 48 | n.a. | n.a. | n.a. | n.a. | 120 | 48 | × | 24 | 360 | n.a. | n.a. | | n.a. | |  |
| Dysarthria | 48 | 48 | 72 | 423 | 48 | n.a. | 120 | n.a. | × | n.a. | × | n.a. | n.a. | | n.a. | |  |
| Dysphagia | n.a. | n.a. | 120 | 423 | n.a. | n.a. | n.a. | n.a. | n.a. | n.a. | × | n.a. | n.a. | | n.a. | |  |
| Tremor, not intentional | n.a. | n.a. | 360 | n.a. | 48 | 72 | n.a. | 48 | n.a. | n.a. | × | n.a. | n.a. | | n.a. | |  |
| Cognitive disabilities | × | × | 0 | 36 | 48 | n.a. | 72 |  | 84 | 96 | × | n.a. | n.a. | | n.a. | |  |
| Delayed speech development | × | 19 | n.a. | 132 | 48 | 0 | n.a. | 24 | n.a. | n.a. | × | n.a. | n.a. | | n.a. | |  |
| Sensory distubances | × | × | 360 | n.a. | n.a. | n.a. | n.a. | × | - | n.a. | 360 | n.a. | n.a. | | n.a. | |  |
| Febrile convulsions | 0-36 | 17/19 | n.a. | × | n.a. | n.a. | n.a. | n.a. | n.a. | n.a. | × | n.a. | n.a. | | n.a. | |  |
| **Malformations** |  |  |  |  |  |  |  |  |  |  |  |  |  | |  | |  |
| Gnathoschisis | n.a. | n.a. | × | × | 0 | n.a. | n.a. | n.a. | n.a. | n.a. | n.a. | n.a. | n.a. | | n.a. | |  |
| Microzephaly | n.a. | n.a. | × | × | 48 | n.a. | 140 | 48 | 84 | 96 | 360 | n.a. | n.a. | | n.a. | |  |
| **Other** |  |  |  |  |  |  |  |  |  |  |  |  |  | |  | |  |
| Hypermobility | n.a. | 19 | × | × | × | n.a. | n.a. | 48 | n.a. | n.a. | n.a. | n.a. | n.a. | | n.a. | |  |
| Ovarian insufficiency | n.a. | n.a. | 276 | n.a. | n.a. | n.a. | n.a. | n.a. | n.a. | n.a. | n.a. | n.a. | n.a. | | n.a. | |  |
| Affective disorder | n.a. | n.a. | 120 | n.a. | n.a. | n.a. | n.a. | n.a. | n.a. | n.a. | n.a. | n.a. | n.a. | | n.a. | |  |
| Urinary disfunction | n.a. | n.a. | 144 | 192 | n.a. | n.a. | × | n.a. | n.a. | n.a. | × | n.a. | n.a. | | n.a. | |  |
| **Medical examinations** | | | | | | | | | | | | | | | | | |
| Full-field ERG | n.a. | n.a. | cone-rod dysfunction | cone-rod dysfunction | rod-cone dysfunction | n.a. | cone-rod dysfunction | cone-rod dysfunction | cone-rod dysfunction | cone-rod dysfunction | cone-rod dysfunction | rod-cone dysfunction | cone-rod dysfunction | | - , foveal atrophy | |  |
| MRT Leukodystrophy | Deep white matter | Deep white matter Periventric. Corpus callosum Capsula interna Middle cerebellar peduncle | Deep white matter Basal ganglia | Subcortical Periventric. | Deep white matter | Post. white matter Capsula interna | Post. white matter Periventric. Parietal lobe Occipital lobe Corpus callosum Capsula Interna Brain stem | Post. white matter Periventric. Parietal lobe Occipital lobe Corpus callosum Capsula Interna Brain stem | Deep white matter Capsula Interna Corpus callosum | Periventricular Corpus Callosum | Capsula Interna Brain stem Upper cerebellar peduncles | Deep white matter | n.a. | | n.a. | |  |
| MRT Brain atrophy | n.a. | n.a. | Cerebrum Cerebellar peduncles Cerebellum Pons Spinal cord | Cerebrum Cerebellum Corpus callosum Brain stem Spinal cord | n.a. | Cerebellum | n.a. | n.a. | n.a. | n.a. | Cerebellum Parietal lobe | n.a. | n.a. | | n.a. | |  |
| MRT Dilatations | n.a. | n.a. | Ventricular system Cisterna prepontina | Sulci Ventricular system | n.a. | n.a. | n.a. | n.a. | n.a. | n.a. | n.a. | n.a. | n.a. | | n.a. | |  |
|  |  |  | |  |  |  |  |  |  |  |  |  |  | |  |  |  |
| **Symbols:** | **×** | **not observed** | | **+** | **elevated** |  |  |  |  |  |  |  |  | |  |  |  |
|  | **n.a.** | **not tested** |  | **/** | **not altered** |  | **Color code for early to late symptom onset** | | | |  |  |  | |  | |  |
|  |  |  |  | **−** | **reduced** |  |  |  |  |  |  |  |  | |  | |  |
|  |  |  |  |  |  |  |  |  |  |  |  |  |  | |  | |  |

**
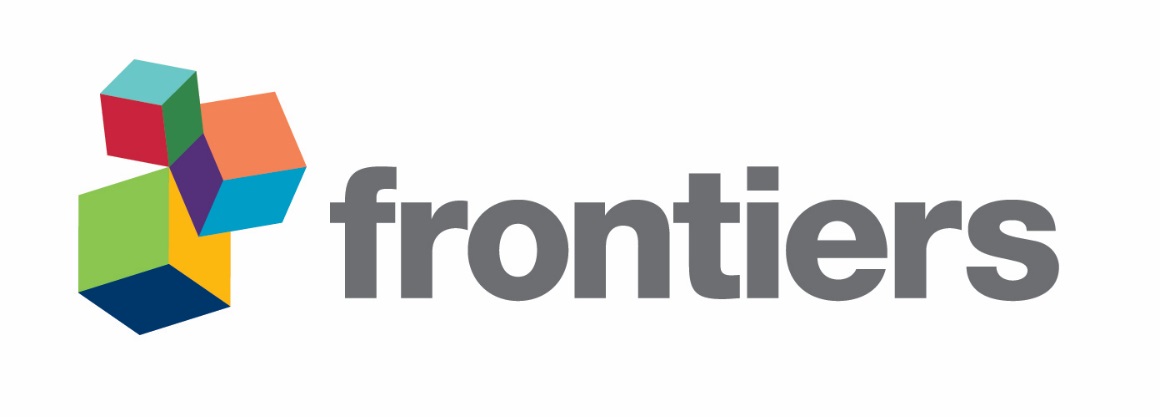
**
